# Correlation between the Frequency of Th17 Cell and the Expression of MicroRNA-206 in Patients with Dermatomyositis

**DOI:** 10.1155/2013/345347

**Published:** 2013-10-30

**Authors:** Xinyi Tang, Xinyu Tian, Yue Zhang, Wei Wu, Jie Tian, Ke Rui, Jia Tong, Liwei Lu, Huaxi Xu, Shengjun Wang

**Affiliations:** ^1^Department of Laboratory Medicine, The Affiliated People's Hospital, Jiangsu University School of Medical Science and Laboratory Medicine, Zhenjiang 212013, China; ^2^Department of Pathology and Centre of Infection and Immunology, The University of Hong Kong, Hong Kong; ^3^Institute of Laboratory Medicine, Jiangsu University, Zhenjiang, China

## Abstract

It was reported that IL-17 had been detected in the inflammatory infiltrates of patients with DM (dermatomyositis). In this study, we investigated the frequency of Th17 cells and the expression of microRNA-206 (miR-206) in DM patients. Firstly, we observed that the frequency of Th17 cells and the expression of transcription factors were increased significantly in the PBMCs of DM patients. Secondly, we found that there was a positive correlation between the percentages of Th17 cells and serum level of CK in DM patients. And the serum concentrations of IL-6, IL-1**β**, TGF-**β**, and IL-23, the important cytokines of Th17 differentiation, were increased in DM patients. It was predicted that Krüppel-like factor 4 (KLF4) is one of the multiple targets of miR-206. We detected the expression of miR-206 in DM patients, and it was decreased in the serum and PBMCs of DM patients. The augmented expression of KLF is accompanied by the attenuated expression of miR-206. Furthermore, a negative correlation between the percentages of Th17 cells and the expression of miR-206 in DM patients has been found. Taken together, these findings suggest the attenuated expression of miR-206, and the augmented frequency of Th17 cells in DM patients.

## 1. Introduction

DM (dermatomyositis) is a subtype of inflammatory myopathies, which is a rare autoimmune disease of skeletal muscle. Patients with DM typically experience the symmetric proximal muscle weakness, electromyographic and muscle alteration, characteristic skin lesion, and elevation of muscle enzymes such as creatine kinase (CK) or lactate dehydrogenase (LDH). Therefore, these clinical symptoms are current diagnostic criteria for DM [1, 2].

Although the pathogenic mechanism of DM is still unclear, it was considered as a CD4+T cells driven disease [3, 4]. On the basis of cytokine expression, CD4+ helper T cells were classified into Th1, Th2, regulatory T cells, and Th17 cells [5]. Early study demonstrated that Th17 cells participate in host defense against extracellular bacteria and fungi. Furthermore, it was observed that Th17 cells are also involved in the process of several inflammatory and autoimmune diseases, such as autoimmune arthritis, Crohn's disease, multiple sclerosis, psoriasis, and Hashimoto's thyroiditis [6–12]. Recent study elucidated that some proinflammatory cytokines such as IL-6, IL-1*β*, TGF-*β*, and IL-23 are critical factors functioning during the process of Th17 cells differentiation [13–16].

 It was reported that IL-17, a key cytokine of Th17 cells, had been detected in the inflammatory infiltrates of patients with DM [17]. And there was a study showed that the concentration of IL-6, a crucial factor of human Th17 cells differentiation, was significantly higher in the serum of DM patients [18]. But there is no study focusing on the alteration of Th17 cells in the peripheral blood of patients with DM. In this study we found the proportion of Th17 cells was augmented in the peripheral blood of patients with DM compared to healthy controls. The expression of RORC gene, a key transcription factor of human Th17 cells, was enhanced in the PBMCs (peripheral blood mononuclear cells) of DM patients. In addition to RORC, Krüppel-like factor 4 (KLF4), a transcription factor of cell differentiation, tumor suppression, stem cell properties, and malignant transformation, is another positive regulator of Th17 differentiation [19–21]. And its expression was enhanced in the PBMCs of DM patients as expected. Also, compared with healthy controls, the level of related cytokines increased in the serum of patients with DM. All the data above suggest that Th17 cells may play a role in the pathogenesis of DM.

MicroRNAs are a class of small noncoding RNAs that modulate gene expression at the posttranscriptional level. They usually bind to the 3′-untranslated regions (UTR) of mRNAs, inhibiting translation, causing accelerated turnover or degradation of the mRNA transcript [22]. It was predicted that KLF4 is one of the multiple targets of miR-206, and an inverse trend between KLF4 and miR-206 has been validated in human [23]. Our study verified that the expression of KLF4 gene was enhanced in the PBMCs, and an attenuated expression of miR-206 was confirmed in the PBMCs as well as the plasma of DM patients. These data suggest that Th17 cells may play a role in the pathogenesis of DM.

## 2. Materials and Method

### 2.1. Individuals and Samples

Twenty-seven patients with DM were included in this study. The diagnosis was based on commonly accepted classification criteria of Bohan and Peter [1]. All the patients were on the period of onset of DM at the time of this study. Main clinical data of these patients are shown in Table 1. Peripheral blood samples were obtained from all patients. The serum concentrations of CK and LDH are abtained by biochemistry test (Beckman au5831, Japan). Thirty healthy control subjects were included, ranging from 32 to 50 years old (42.73 ± 5.058, data correspond to the arithmetic mean ± SD of the ages of the control subjects).

All samples were taken in accordance with the regulations and approval of the Affiliated People's Hospital of Jiangsu University.

### 2.2. Cell Isolation and Stimulated In Vitro

Peripheral blood mononuclear cells (PBMCs) were isolated by density-gradient centrifugation over Ficoll-Hypaque solution. PBMCs were washed and incubated in complete RPMI 1640 culture medium in the presence of 50 ng/mL phorbol myristate acetate (PMA; Sigma-Aldrich St. Louis, MO) and 1.0 *μ*g/mL ionomycin (Sigma-Aldrich) for 5 h. After 5 h of culture at 37 Cunder 5% CO_2_, cells were collected and centrifuged for Th17 cells and mRNA detection by flow cytometric analysis and qRT-PCR, respectively.

### 2.3. Flow Cytometric Analysis

Stimulated PBMCs were washed and immunostained with phycoerythrin-cy5-conjugated anti-CD3 (eBioscience, San Diego, CA), fluorescein isothiocyanate-conjugated anti-CD8 (eBioscience, San Diego, CA), and phycoerythrin- (PE-) conjugated anti-IL-17 (eBioscience, San Diego, CA) mAb against human cells. Isotype-matched Ab controls were used in all procedures. All the staining was according to manufacturers' protocol. Cells were analyzed using a FACSCalibur flow cytometer and CELLQUEST software (Becton Dickinson, Sparks, MD), and results were expressed as the percent of CD3+CD8− cells expressing IL-17.

### 2.4. RNA Isolation and Real-Time PCR

For the detection of cytokine IL-17, transcription factor RORC, and KLF4, PBMCs were stimulated for 5 h as described above. After that activated cells were used to quantify the expression of IL-17 and RORC mRNA by real-time PCR.

TRIzol reagent (Invitrogen, Carlsbad, CA) was added in stimulated PBMCs. After isolated total RNA, reverse transcription was performed according to the manufacturer's instruction (Toyobo, Osaka, Japan). MicroRNA in plasma was isolated with NucleoSpin:emoji: miRNA Plasma (Macherey-Nagel, Düren, German). miRNA qRT-PCR Primer Set (Ribo, Guangzhou, China) and M-MLV Reverse Transcriptase (Takara, Dalian, China) were used for miR-206 and U6 reverse transcription.

Real-time PCR was performed in duplicate using Bio-Rad SYBR green super mix (Bio-Rad, Hercules, CA). Primer sequences were as follows: IL-17, sense, 5′-CAAGACTGAACACCGACTAAG-3′; antisense, 5′-TCTCCAAAGGAAGCCTGA-3′, RORC, sense, 5′-CCTGGGCTCCTCGCCTGACC-3′; antisense, 5′-TCTCTCTGCCCTCAGCCTTGCC-3′, KLF4, sense, 5-CAA GTC CCG CCG CTC CAT TAC CAA-3; anti-sense, 5-CCA CAG CCG TCC CAG TCA CAG TGG-3., miR-206, sense, 5-GAGTGCTGGAATGTAAGGAAG-3; antisense, 5-GCAGGGTCCGAGGTATTC-3.

 We used *β*-actin or U6 as internal control. Data were analyzed by Bio-Rad CFX Manager software.

### 2.5. Cytokine Quantification

Levels of IL-6, IL-1*β*, TGF-*β*, and IL-23 were determined by ELISA (eBioscience, San Diego, CA) using an ELISA reader (*μ*Quant, BIO-TEK, USA). All determinations were performed in duplicate, and the lower detection limits for IL-6, IL-1*β*, TGF-*β*, and IL-23 were 1.5625, 1.5625, and 125 pg/mL, 15.625 pg/mL, respectively.

### 2.6. Statistical Analysis

Student's unpaired or paired *t* test was performed to determine whether there was a statistically significant change between two groups. Correlations between variables were determined by Spearman's correlation coefficient. Data were analyzed with GraphPad Prism5 software (GraphPad Software, Inc., San Diego, CA).

## 3. Results

### 3.1. Th17 Lymphocytes in the PBMCS of Patients with DM

**Figure 1:**
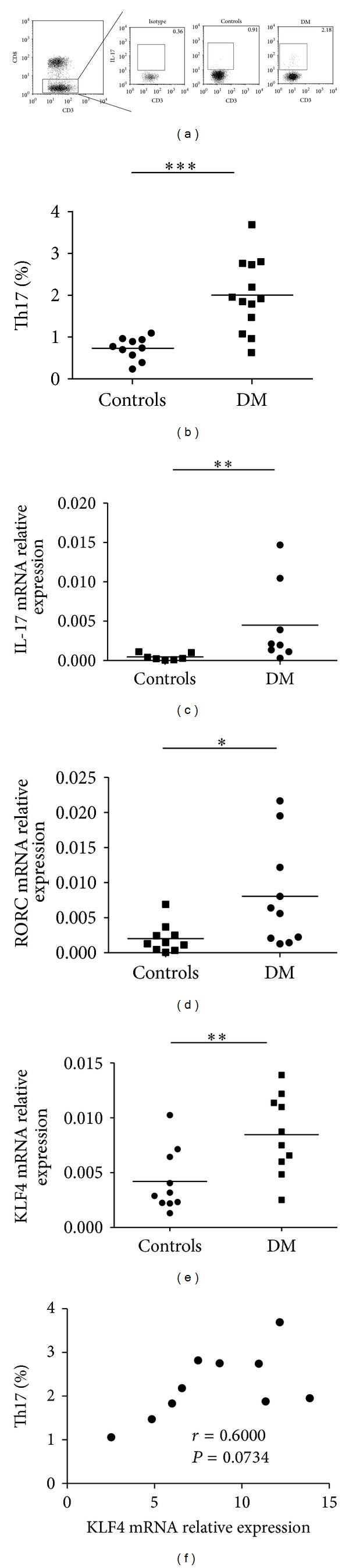
Th17 cells in the peripheral blood from DM patients. PBMCs from DM patients and healthy controls were incubated with PMA/ionomycin, stained for cell surface molecules CD3 and CD8 as well as intracellular IL-17, and analyzed by flow cytometry. (a) Representative dot plots from DM patient and a control subject are shown. Values correspond to the percentage of Th17 lymphocytes. We used isotype controls to determine the positive cells, and all the values are gated on the CD3+CD8− cells. (b) Percentages of Th17 lymphocytes were compared between DM patients and control subjects. (c) Levels of IL-17 mRNA in PBMCs pretreated with PMA/ionomycin from DM patients and controls. (d) The levels of RORC mRNA in PBMCs were detected by real-time PCR from DM patients and healthy controls. (e) The levels of KLF4 mRNA in PBMCs were detected by real-time PCR from DM patients and healthy controls. (f) The correlation between the percentages of Th17 lymphocytes and the expression of KLF4 mRNA in PBMCs from DM patients. Each data point represents an individual subject; horizontal lines show the mean. *P < 0.05; **P < 0.01; ***P < 0.001.

To quantify Th17 cells in peripheral blood of patients with DM; first we analyzed the proportion of CD3+CD8−IL-17+lymphocytes in PBMCs of DM patients by flow cytometry. The expression of CD4 molecule on PBMCs is downregulated after being stimulated with PMA and ionomycin, so we gated on CD3+CD8− in PBMCs and identified IL-17+cells to distinguish the Th17 cells from activated T cells in PBMCs (Figure 1(a)). The percentage of Th17 cells was significantly increased in PBMCs from patients with DM compared with healthy controls (*P* = 0.0007, Figure 1(b)). 

qRT-PCR analysis displays an enhanced expression of IL-17mRNA in the PBMCs from DM patients, with lower expression in healthy controls (Figure 1(c)).

To further document the state of Th17 cells in DM patients, we analyzed the expression of RORC and KLF4 mRNA, which plays a considerable role in differentiation of Th17 cells. As expected, the expression of RORC or KLF4 mRNA in PBMCs from DM patients is significantly higher than that from healthy controls (Figures 1(d) and 1(e)). In addition, we found high levels of KLF4 mRNA expression with increased circulating Th17 lymphocytes in DM patients, and there was a modest correlation between the percentages of Th17 cells and the expression of KLF4 mRNA in PBMCs from DM patients (*r* = 0.6000, *P* = 0.0734) (Figure 1(f)). 

These results collectively indicate that the proportion of Th17 cells in DM patients is augment. 

### 3.2. High Levels of CK with Increased Circulating Th17 Cells in DM Patients

**Figure 2:**
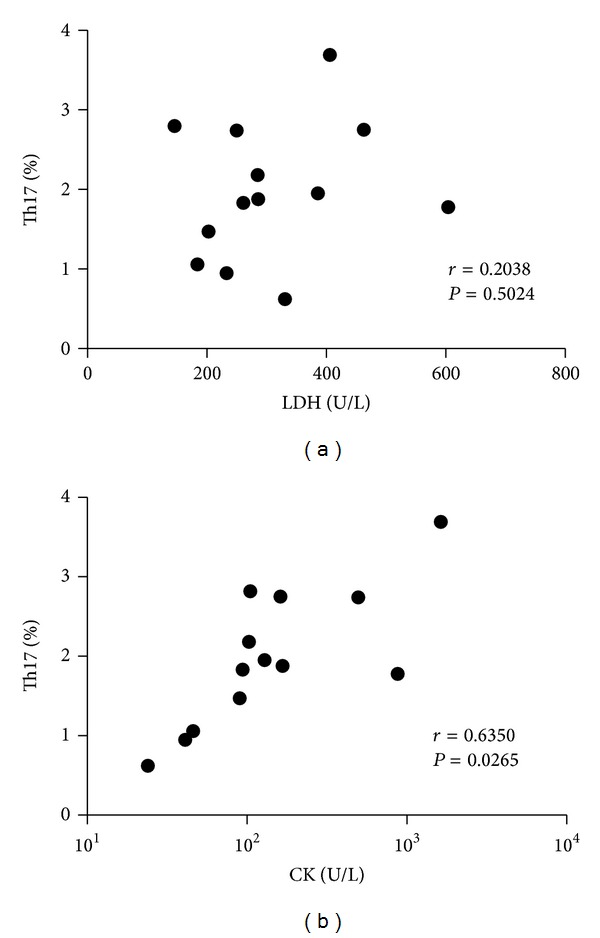
The correlation between the percentages of Th17 lymphocytes in PBMCs and LDH (left)/CK (right) in DM patients.

In patients with DM, the muscle enzymes such as CK or LDH are upregulated, and they are index of prognosis for DM. So we analyzed the correlation between clinical and laboratory parameters. And we found that there was a positive correlation between the percentages of Th17 cells and serum level of CK (*r* = 0.6350, *P* = 0.0265), but not with the level of LDH (*r* = 0.2089, *P* = 0.5147) (Figure 2). This result demonstrated that the percentage of Th17 cells could reflect the severity of DM in some extent.

### 3.3. Serum Levels of Cytokines Related to Th17 Lymphocytes Differentiation

**Figure 3:**
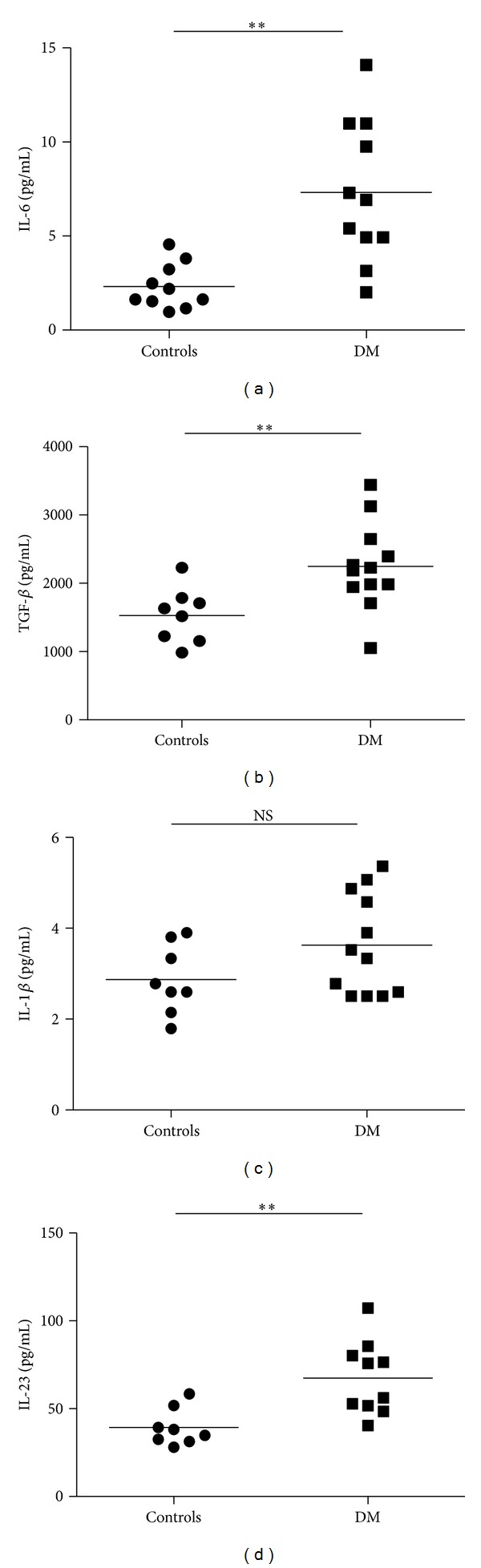
Serum levels of proinflammatory cytokines are increased in DM patients compared with healthy controls. (a) Serum levels of IL-6 were determined by ELISA in serum samples from DM patients and controls. (b) Serum levels of IL-1*β* were determined by ELISA in serum samples from DM patients and controls. (c) Serum levels of TGF-*β* were determined by ELISA in serum samples from DM patients and controls. (d) Serum levels of IL-23 were determined by ELISA in serum samples from DM patients and controls. Horizontal lines show the mean. **P < 0.01; ns: no significant differences.

It was reported that IL-6 and IL-1*β* are essential in the differentiation of Th17 cells, and some investigators considered that TGF-*β* facilitates the differentiation of Th17 cells. In order to elucidate the influencing factors of Th17 cells enhancement in DM patients, we analyzed the levels of IL-6, IL-1, TGF-*β*, and IL-23 in serum from DM patients and healthy controls. We found that DM patients have significantly increased serum concentration of IL-6, TGF-*β*, and IL-23 in comparison with healthy controls (*P* = 0.0011, *P* = 0.0097, and *P* = 0.0031, resp., Figures 3(a), 3(b), and 3(d)). In addition, serum concentrations of IL-1*β* are higher in DM patients compared with healthy controls, but this difference did not reach statistical significance (Figure 3(c)). 

 These data suggest that the high level of these related cytokines may promote the augmentation of Th17 cells in DM patients.

### 3.4. Attenuated miR-206 Expression in Patients with DM

**Figure 4:**
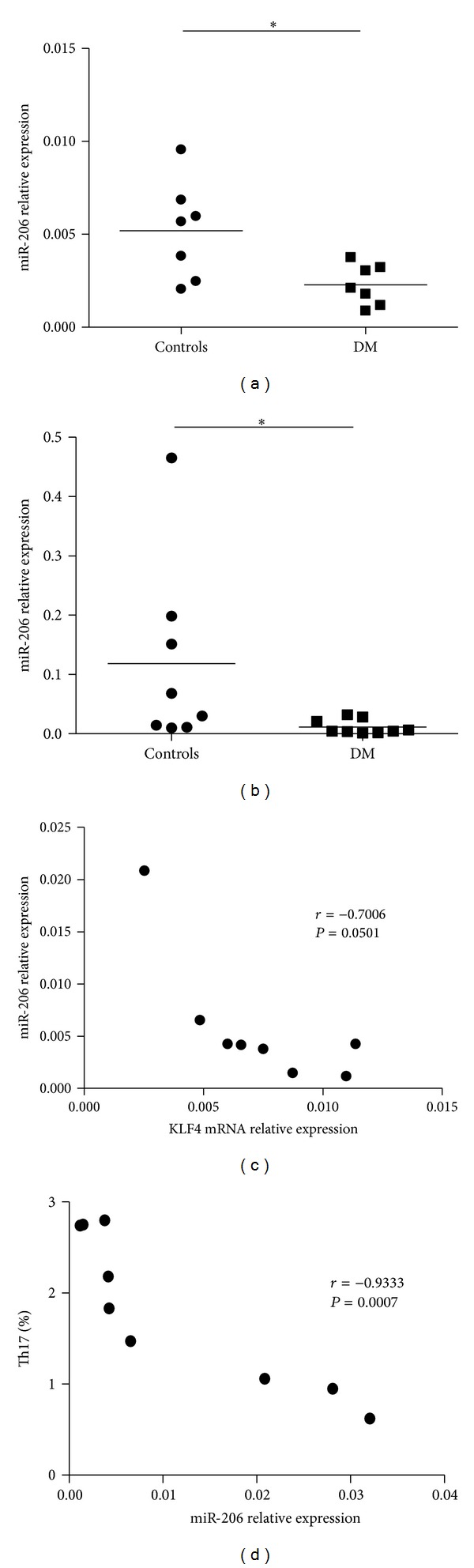
Levels of miR-206 were decreased in DM patients compared with healthy controls. (a) Serum levels of miR-206 were determined by real-time PCR in serum samples from DM patients and controls. (b) The level of miR-206 in PBMCs was detected by real-time PCR from DM patients and controls. (c) The correlation between the expression of miR-206 and the expression of KLF4 mRNA in DM patients. (d) The correlation between the percentages of Th17 lymphocytes in PBMCs and the expression of miR-206 in DM patients. Horizontal lines show the mean. *P < 0.05.

KLF4 plays a considerable role in differentiation of Th17 cells. We have certified the augment expression of KLF4 mRNA in PBMCs of DM patients, and we also found that there was a modest correlation between the percentages of Th17 cells and the expression of KLF4 mRNA. The inverse trend between KLF4 and miR-206 has been validated in a previous study [16], so we detected the expression of miR-206 in the serum and PBMCs of DM patients by qRT-PCR. As a result, the expression of miR-206 is significantly attenuated in the PBMCs and serum of DM patients as compared to healthy controls (Figures 4(a) and 4(b)). Also, there was a modest correlation between the expression of miR-206 and the expression of KLF4 mRNA (*r* = − 0.7006, *P* = 0.0501) (Figure 4(c)). Apart from this, a negative correlation between the percentages of Th17 cells and the expression of miR-206 in PBMCs of 9 DM patients has been found in this study (*r* = − 0.9333, *P* = 0.0007) (Figure 4(d)). 

These findings suggest that the augmented expression of KLF4 mRNA may be caused by the attenuated expression of miR-206, and the high level of KLF4 mRNA evokes the proportion of Th17 cells in DM patients.

## 4. Discussion

Dermatomyositis (DM) is an autoimmune myopathies characterized by common muscle weakness and an inflammatory infiltrate in muscle [1]. The conventional and available treatment of DM patients is intravenous corticosteroid and immunosuppressive agent. As it is known, long-term use of corticosteroid and immunosuppressive agent would impact the immunological balance in patients. But the pathogenesis of DM is still unclear, and the investigation in this aspect is limited. Therefore, identification of the involved mechanisms may provide an important step towards developing therapeutic strategies for DM patients. 

DM was considered as a CD4+T cells driven disease [4]. CD4+T cells were defined as Th1, Th2, Treg, and Th17 based on the profiles of cytokine secretion [5]. It has been demonstrated that Th17 cells were involved in several autoimmune diseases. A recent study showed that IL-17-producing cells have been detected in muscle biopsy from DM patients [17]. And the level of IL-6, the important factor of Th17 cells differentiation, increased in serum of DM patients compared with healthy controls [18]. We speculated that there could be an alteration of Th17 cells in the peripheral blood of patients with DM. To confirm this hypothesis, we assessed the proportion of Th17 cells and the expression of IL-17 mRNA in the peripheral blood of 13 patients with DM and 10 healthy controls. We identified CD3+CD8−IL-17+ cells to distinguish the Th17 cells from PBMCs. As expected, increased CD3+CD8−IL-17+ cells and enhanced expression of IL-17 mRNA were found in PBMCs from patients with DM. 

Recent researches have demonstrated that RORC is a critical transcription factor of human Th17 cells needed to orchestrate the differentiation of human Th17 cells [24]. Our study has found that there was an enhanced expression of RORC mRNA in PBMCs from DM patients compared with control subjects. And we also found that the expression of KLF4, another critical positive regulator of Th17 differentiation [20], was increased in PBMCs from DM patients. The data exhibit the enhancement of Th17 cells on mRNA level.

It has been widely accepted that the upregulation of muscle enzymes such as CK and LDH is the most objective clinical manifestations of DM; it could evaluate the development of DM and show the prognosis for this disease [25]. We analyzed the correlation between proportion of Th17 cells and level of muscle enzyme. It was shown that there was a positive correlation between the percentages of Th17 cells and serum level of CK, but not with the level of LDH. These results collectively indicate that the proportion of Th17 cells in DM patients is augmented and could reflect the severity of DM to some extent.

To investigate the influential factor of Th17 cells augmentation in DM patients, we detected the proinflammatory cytokines which are essential for the human Th17 differentiation. In previous researches, IL-6 and IL-1*β* were regarded as the key factors of human Th17 differentiation [14, 15]. But the standpoint about the function of TGF-*β* in human Th17 differentiation is still controversial. Some investigators considered that TGF-*β* facilitated the differentiation of Th17 cells [16], but others sustained the negative function of TGF-*β* in Th17 differentiation [14]. We found that DM patients had significantly increased serum concentration of IL-6 and TGF-*β* in comparison with healthy controls, and serum concentration of IL-1*β* is higher in DM patients compared with healthy controls, but this difference did not reach statistical significance. These results demonstrated that the augmentation of Th17 cells in DM patients may be influenced by the high level of these proinflammatory cytokines.

To further analyze the influential factor of Th17 cells enhancement in DM patients, we considered searching the miRNA which may influence the enhancement of Th17 cells. KLF4 is one of the targets of miR-206, and an inverse relationship between miR-206 and KLF4 in human has been confirmed [23]. After acquiring the increased expression of KLF4 mRNA in PBMCs of DM patients, we verified the decreased expression of miR-206 in PBMCs and serum of DM patients. Apart from these, we found that there was a high level of KLF4 expression with decreased miR-206 expression in PBMCs of DM patients, and the correlation between expression of KLF4 and miR-206 in DM patients' PBMCs was modest. Moreover, there was a negative correlation between the percentages of Th17 cells and the expression of miR-206 in PBMCs of DM patients. These data manifested that the augmented expression of KLF4 mRNA may be caused by the attenuated expression of miR-206, and the high level of KLF4 mRNA evokes the proportion of Th17 cells in DM patients. 

In summary, our data collectively suggest that there is an augmented frequency of Th17 lymphocytes in DM patients, and the attenuated expression of miR-206 may regulate the percentage of Th17 cells in DM patients to some extent.

## Figures and Tables

**Table 1:** Clinical features of DM patients included in the study.

	DM	Range
*n *	28	
Gender (M/F)	8/20	
Age (yr)	52.29 ± 14.09	27–59
LDH (U/L)	438.6 ± 277.7	146.0–1287
CK (U/L)	864.2 ± 1460	24.00–5694

Data correspond to the arithmetic mean ± SD, M: male; F: female.
